# Insights into mechanism kinematics for protein motion simulation

**DOI:** 10.1186/1471-2105-15-184

**Published:** 2014-06-12

**Authors:** Mikel Diez, Víctor Petuya, Luis Alfonso Martínez-Cruz, Alfonso Hernández

**Affiliations:** 1 Faculty of Engineering in Bilbao, University of the Basque Country UPV/EHU, Department of Mechanical Engineering, Alameda de Urquijo s/n, 48013 Bilbao, Spain; 2 CIC bioGUNE Research Centre, Ed. 800 Parque Tecnolgico de Bizkaia, 48160 Derio, Spain

**Keywords:** Protein, Kinematics, Simulation, Secondary structure detection

## Abstract

**Background:**

The high demanding computational requirements necessary to carry out protein motion simulations make it difficult to obtain information related to protein motion. On the one hand, molecular dynamics simulation requires huge computational resources to achieve satisfactory motion simulations. On the other hand, less accurate procedures such as interpolation methods, do not generate realistic morphs from the kinematic point of view. Analyzing a protein’s movement is very similar to serial robots; thus, it is possible to treat the protein chain as a serial mechanism composed of rotational degrees of freedom. Recently, based on this hypothesis, new methodologies have arisen, based on mechanism and robot kinematics, to simulate protein motion. Probabilistic roadmap method, which discretizes the protein configurational space against a scoring function, or the kinetostatic compliance method that minimizes the torques that appear in bonds, aim to simulate protein motion with a reduced computational cost.

**Results:**

In this paper a new viewpoint for protein motion simulation, based on mechanism kinematics is presented. The paper describes a set of methodologies, combining different techniques such as structure normalization normalization processes, simulation algorithms and secondary structure detection procedures. The combination of all these procedures allows to obtain kinematic morphs of proteins achieving a very good computational cost-error rate, while maintaining the biological meaning of the obtained structures and the kinematic viability of the obtained motion.

**Conclusions:**

The procedure presented in this paper, implements different modules to perform the simulation of the conformational change suffered by a protein when exerting its function. The combination of a main simulation procedure assisted by a secondary structure process, and a side chain orientation strategy, allows to obtain a fast and reliable simulations of protein motion.

## Background

To address functional requirements or interact with other biological molecules, proteins undergo structural changes of variable degree, varying between distinct overall conformations, of which only some are usually determined experimentally (i.e the activated and inactivated forms of an enzyme). This is caused by the difficulties in the obtention of X-ray quality diffracting crystals, and consequently, it limits the knowledge on the dynamic behavior of the biomolecular machinery in important biological processes. Accordingly, the comprehension of the intermediate steps is crucial to overcome these difficulties, and provide a useful tool to fill the gaps that escape to bench-dependent experimental approaches so far. However, protein motion simulation has always been a troublesome problem, mostly because of its high demanding computational requirements. Precise simulations based on molecular dynamics are usually limited to small molecules or to the use of supercomputers or distributed networks [1-3]. However, other procedures such as Ab initio or Rosetta methods do not provide information related to protein kinematics. This information is essential if we want to understand the mechanisms that proteins use to exert their motion and hence, their functions [4].

Recently, and thanks to the information available related to protein science, new approaches have been proposed in the literature [5,6] to simulate protein motion. These approaches are not based on either quantum mechanisms, or biology related roots, but deal with mechanism and robot kinematics principles [5]. One of the main advantages of these new methodologies is their small computational cost. One of the first applied methods is Probabilistic Roadmap Method (PRM) [6-11]. This method consists in discretizing the protein configurational space. Then, each position is evaluated against a scoring function (force field, empirical, etc) and it is considered either correct or incorrect. Once every position has been checked it is possible to trace a path connecting neighboring positions to obtain the protein motion. The PRM is used on a wide variety of protein motion studies. In [12,13] it is proposed to use this approach to the simulation of ligand-protein interaction. In those works, it is proposed to consider the degrees of freedom of the ligand, as well as some degrees of freedom of the protein (mostly side chains related degrees of freedom). In [6], a similar approach, considering only the ligand degrees of freedom, is proposed. In [14], it is studied how restrictions to the possible motions of the protein backbone affect the search algorithms used for PRM. Although this approach yields quite good results, the need of computing all possible configurations of the protein structure makes this procedure computationally costly, especially for big proteins.

One important approach used for protein simulation, is related to the Normal Mode Analysis (NMA) implemented in mechanisms and robotics. This analysis provides information related to vibrational modes of mechanisms, useful for the dynamic analysis of their structure [15]. This approach, computationally much less expensive than PRM, may be applied to protein structures [16,17]. Using this methodology, information related to possible movements of a protein structure around its current configuration is obtained. Thus, although computationally less expensive, the information provided is not complete. New procedures combine both NMA and PRM to obtain large conformational changes in proteins [18]. In this approach NMA results are used to guide the PRM algorithms and reduce their computational cost. Kinetostatic compliance method [19,20] makes use of several kinematic theories to simulate protein motion. Firstly, it takes advantage of zero notation [21] to simplify protein structure definition during the procedure. Secondly, It also implements ball and rods modelization, considering both bond lengths and angles as constant. To execute the simulation process the protein potential energy field is transformed into equivalent forces and torques applied to the protein chain. Basing on the applied torques, it calculates the dihedral angle increments for the next step of the simulation.

In this paper, we present a new methodology based on our previous works [22,23] for protein motion simulation. The objective of the procedure is to morph a protein from one known configuration to another known one, providing reliable and quick information in relation to protein kinematics and movement with a very low computational cost, (low enough to be used on a normal PC). The simulation procedure presented in this paper is composed of four independent strategies. The first one consists in a normalization procedure aimed to homogenize equivalent bond distances and angles in the protein structure [22]. The second one is a main simulation procedure entrusted to advance in the simulation obtaining valid structures [23]. A third strategy is a procedure intended to reduce protein’s potential energy by changing side chains orientation. And the final one detects secondary structures among the protein chain. The novelty of the proposed procedure consists, on the one hand, in the implementation of the side chain orientation strategy and the secondary structure detection method, and on the other hand, in the simultaneous combination of the aforementioned four strategies. Consequently, the approach provides a computationally efficient simulation tool for protein motion simulation. To validate the results, three indicators are measured through the simulation process: (i) backbone atom root mean square deviation to compare obtained structures global similarity, (ii) Ramachandran plots, to ensure proteins biological nature and (iii) protein’s potential energy to verify that no steric clashes have occurred during the simulation [24].

## Methods

### Preparing protein structure for kinematics modeling

Ball and rods models provide model structures valid to apply mechanism theorems for protein simulation [21]. Most protein structure models whose target is protein simulation incorporate some simplifications. *Cα* meshes are used in [25-27] to produce a reduced model with an acceptable computational cost. Rigid bond and angle approaches are used in the same way in [28,29]. Normal mode analysis (NMA), usually mixes rigid bonds with springs to produce the structure needed for modal analysis [17,30]. Side chains are also simplified in various ways to reduce the computational cost associated to them. In [8] it is proposed to treat side chains as spheres filling an equivalent space. In [28], the authors propose to adjust the size of side chains to reduce its influence on the simulation, and later on, resize them progressively.

In this paper we propose an all atom model, based on ball and rods approach, in which some simplifications are considered to reduce the overall computational cost. In particular, Protein degrees of freedom are reduced to rotations around the dihedral angles. Every other possible atom movement resulting from bond stretching or non-proper torsions is despised. Besides the relative position among peptide plane atoms is maintained constant during the simulation process. The peptide bond angle *ω* value is also limited to 0° or 180°. Regarding side chains, according to the proposed all atom model, every atom should be taken into account. In relation to side chains’ degrees of freedom, to reduce the computational cost of the process, only the rotation around the *Cα*-*Cβ* bond is considered.

As previously stated, the procedure simulates protein motion between two known structures. Most structural data for protein simulation come from experimentation. Thus, these data need to be compared with the proposed model to verify that all the hypotheses are fulfilled. One of the major drawbacks of trying to use kinematic theories to protein simulation is the difference between proteins and mechanisms structures. In mechanism kinematics the linkages do not change their structural form or characteristics during the movement, unless the objective of the mechanism lies precisely in that requisite [31]. However we must bear in mind that proteins are composed by atoms that atoms that are bonded by electromagnetic and covalent forces, thus, in the case of proteins, there is no need to maintain constant neither bond length nor bond angles during the conformational change.

Therefore a normalization procedure is applied to experimental data with the purpose of homogenizing equivalent bond distances and angles in the protein structure. The approach is based on two different normalization processes, peptide plane normalization and bond length normalization.

Peptide planes are normalized to get exactly 0° or 180° peptide bond angles. This complies with the proposed hypothesis of rigid peptide planes among the simulation [23]. The objective of normalizing the peptide planes is to assign to *ω*_*i*_ the angles 0° or 180°, as it is proposed in the kinematic model. To do this, *Cα*_*i*_ and *Cα*_*i*+1_ atoms’ coordinates are fixed. Then, using the least square method, the mean plane is calculated using the local coordinates of *Cα*_*i*_,*C*_*i*_,*O*_*i*_,*N*_*i*+1_ and *Cα*_*i*+1_ atoms. Fixing *Cα*_*i*_ and *Cα*_*i*+1_ atoms’ coordinates allows to maintain protein backbone continuity during the normalization process, as the normalization of one peptide plane does not alter the one that has been already normalized.

Local reference systems to determine relative locations of amino acids atoms have also been used in [32,33]. Here, *Cα*_*i*_ is selected as the origin of the local coordinate system. The straight line connecting *Cα*_*i*_ and *Cα*_*i*+1_ defines **u**_*i*_ axis direction. **w**_*i*_ axis direction is defined by the perpendicular direction to the plane containing *Cα*_*i*_ and *C*_*i*_ atoms and **u**_*i*_ axis. Finally, **v**_*i*_ completes the right-handed reference system. The advantage of using this local reference system, is that the middle plane will always contain **u**_*i*_ axis, so the least square problem is reduced to the calculus of the slope of this plane. This is made by the following formula:

(1) $$S={\left(w_{C_{i}}\;-\;a\cdot v_{C_{i}}\right)}^{2}\;+\;{\left(w_{O_{i}}\;-\;a\cdot v_{O_{i}}\right)}^{2}\;+\;{\left(w_{N_{i+1}}\;-\;a\cdot v_{N_{i+1}}\right)}^{2}$$

where *v*_*j*_,*w*_*j*_ are the local coordinates of *C*_*i*_,*O*_*i*_,*N*_*i*+1_ atoms and *a* is the slope of the plane to be calculated. To find the value of *a*, Eq. (1) is differentiated with respect to *a* obtaining the result in Eq. (2):

(2) $$a=\frac{w_{C_{i}}\cdot v_{C_{i}}+w_{O_{i}}\cdot v_{O_{i}}+w_{N_{i+1}}\cdot v_{N_{i+1}}}{v_{C_{i}}^{2}+v_{O_{i}}^{2}+v_{N_{i+1}}^{2}}$$

**Figure 1:**
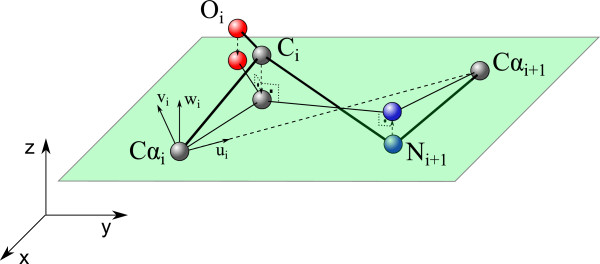
Normalization of the peptide planes.

Once the slope of the plane has been defined, atoms *C*_*i*_, *O*_*i*_ and *N*_*i*+1_ are projected onto it (Figure 1).

In a second stage, distance constraints are applied to ensure that every bond length is identical between two same type of atoms. To apply these distance constraints, we need to define standard bond lengths. For this matter, reference bond lengths from the AMBER force field are used [34]. Distance constraints are sequentially applied from the fist atom of the protein chain to the last one. To apply the distance constraint, the formulation proposed in [35,36] is used. Thus, using *j* as a reference atom, the distance constraint is applied to the bond between *j* and *i* atoms according to the following expressions:

(3) $$\begin{array}{ll} \left|r_{i}^{1}-r_{j}^{1}\right| & =d_{\text{ji}}\; \end{array}$$

(4) $$\begin{array}{ll} \left(r_{i}^{0}-r_{j}^{1}\right)\times \left(r_{i}^{1}-r_{j}^{1}\right) & =0\; \end{array}$$

where $r_{j}^{1}$ and $r_{i}^{0}$ represent the position vectors of the atoms *j* and *i* respectively before the application of the corresponding distance constraint, whereas $r_{i}^{1}$ is the vector determining the new position of atom *i*. *d*_*j* *i*_ defines the theoretical mean value of the bond length. Once the distance constraint has been applied to *i* atom, every subsequent atom of the protein chain is translated by $\left(r_{i}^{1}-r_{i}^{0}\right)$.

**Figure 2:**
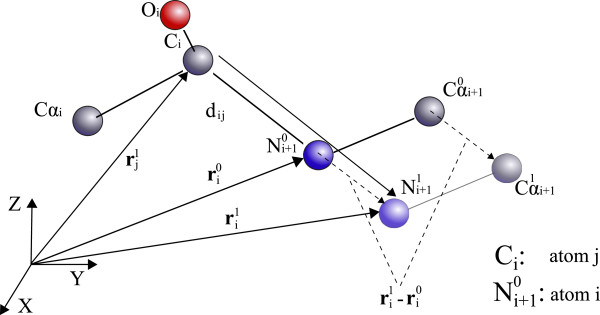
Application of the distance constraint to bond lengths.

As Eq. (3) defines a sphere of radius *d*_*j* *i*_ it is necessary another constraint to univocally determine the position of *i* atom. This new condition determines that the new position fo *i* atom must lie on the straight line defined by the actual positions of *i* and *j* atoms. In Figure 2, the application of the distance constraint to *C*_*i*_ (*j* atom) and *N*_*i*+1_ (*i* atom) is represented.

To test the viability of the normalization process, Type-C PPase protein from S. gordonii is used, which is supposed to suffer a conformational change from a “closed” (PDB 1k20) to an “open” (PDB 1k23) structure upon binding of pyrophosphate (PPi). SgPPase exists as an homodimer in solution, in which each subunit is composed of two distinct domains. The N-terminal domain consists of a six-stranded parallel b-sheet flanked by extended loops on one side and a helical subunit on the other one. In the absence of ligands, the C-terminal domains occlude the corresponding active sites. Binding of PPi or substrate analoges to the active site of the N-terminal domain presumably triggers a conformational change that liberates the inhibition imposed by the C-terminal region [37]. Three strategies have been tried, applying distance, planes and sequential (planes and next distance) normalization processes. The results for the normalization of the initial and final reference conformations of the movement are shown in the Table 1 where they are compared against the protein before the normalization.

**Table 1:** Results of the normalization processes

**Normalization**	***rmsd***_***l ***_**(*Å*)**		**Energy (Kcal/mol)**		**RP (atoms in preferred regions)**	
	**1k20**	**1k23**	**1k20**	**1k23**	**1k20**	**1k23**
Lengths	0.36	0.18	-12.6*%*	-13.7*%*	94*%*	92*%*
Planes	0.17	0.08	3*%*	1*%*	92*%*	91*%*
Sequential (P+L)	0.37	0.21	-10*%*	-8.6*%*	93*%*	91*%*

Table 1 shows that the normalization processes have not altered the global structure of the proteins, the higher root mean square deviation being only 0.37 *Å*. Regarding potential energy variation, length normalization process reduces its value in both 1k20 and 1k23 proteins. However, the proteins’ potential energy value is increased by 3*%* on 1k20 protein and by 1*%* on 1k23 protein on the planes normalization process. This effect is the result of projecting the atoms onto the calculated middle plane, producing the bonds distances change. Finally, sequential normalization process reduces this effect by first applying peptide plane normalization and next length normalization. As stated before, every distance constraint displaces the subsequent atoms of the protein chain, thus not altering already normalized peptide planes. In every normalization process, Ramachandran plot values indicate that the normalized protein structures have always maintained their biological meaning. After analysing these results, from these three normalization processes the sequential one has been chosen. This normalization process fits the experimental data with the proposed model of the protein structure. Additionally, the existence of normalized peptide planes allows to exclude the peptide bond angle *ω* as a degree of freedom.

### Simulation procedure for dynamic dihedral angle increments adjustment

The main target of the methodology described in this paper is to simulate the protein motion between two known positions. To that aim, we have simulated the conformational changes suffered by three different proteins, (i) Type-C Inorganic Pyrophosphatases; (ii) Troponin C; and (iii) Calmodulin, ranging from the displacement of an entire domain to the reorientation of few secondary structure elements. The proposed model of the protein structure permits considering only the dihedral angles as proteins degrees of freedom to produce the motion. This configuration resembles the protein with a very long serial robot. Thus, the motion of the protein can be defined as a sequence of incremental steps applied on these dihedral angle values, from the initial to the final conformations. Data structures of the proteins under study are taken from the Protein Data Bank (PDB), which are used as input data for the procedure. The simulations were carried out using a software developed by our research group called GIMPRO [38]. Several options are implemented in the software as it is stated subsequently. The software is able to read and save protein data with *.pdb* file format. It also produces text files with the evolution of both studied protein’s potential energy and rmsd evolution. Finally the software creates a *.pdb* file containing the obtained simulation. This file is compatible with other visualization programs like PYMOL in order to produce higher quality renderizations of the protein movement.

The process uses dihedral angles increment data, *Δϕ*_*i*_ and *Δψ*_*i*_, to complete the simulation. This data is obtained by calculating dihedral angle values of the final conformation $\left(\phi_{i}^{f}\;\text{and}\;\psi_{i}^{f}\right)$ and subtracting dihedral angle values of the initial conformation $\left(\phi_{i}^{0}\;\text{and}\;\psi_{i}^{0}\right)$ as stated on Eq. (5). The total increment in each dihedral angle is divided by *p*, the number of intermediate conformations to be computed. For each dihedral angle, the direction that requires the minimum angular increment to reach the final position is defined as its starting rotation direction.

(5) $$\begin{array}{l} \Delta \phi_{i}=\frac{\phi_{i}^{f}-\phi_{i}^{0}}{p};\Delta \psi_{i}=\frac{\psi_{i}^{f}-\psi_{i}^{0}}{p} \end{array}$$

To assess the quality of the obtained structures, root mean square deviation (rmsd), Ramachandran plots [39] and potential energy will be evaluated during the simulation process. Regarding the root mean square deviation, backbone atoms are considered for its calculus. To evaluate proteins’ potential energy, AMBER potential force field, with the parameters proposed by Cornell [34] has been chosen. The use of the three indicators ensures the global similarity between the structures (rmsd), the non-existence of steric clashes (potential energy) and the biological sense of the obtained structures (Ramachandran plots). Intermediate data structures for rmsd comparison are obtained from the Morph server [40].

**Figure 3:**
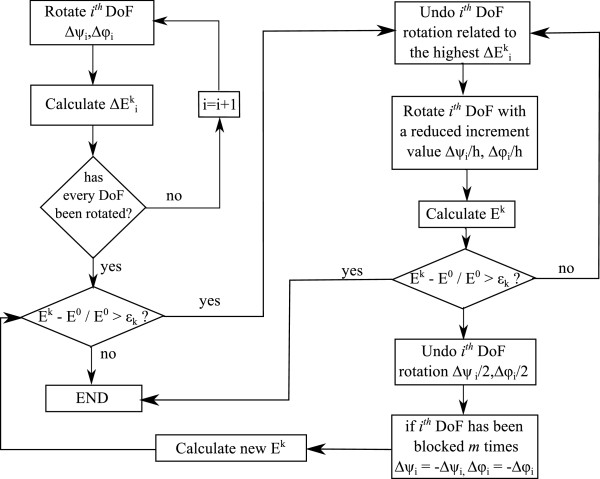
**Algorithm for the *k*-th step of the procedure.**

In Figure 3 the flux diagram of the simulation procedure is shown. The first step is to calculate protein’s initial conformation potential energy *E*^0^. To obtain the next protein’s conformation, the procedure starts rotating the dihedral angles of the protein in sequential order, from the first amino acid to the last one. One important characteristic of the process is that it stores the increment produced by each rotation on the protein’s potential energy as $\Delta E_{i}^{k}$, where *k* is the actual step number. Once the process has finished the actual step’s proteins’ potential energy is obtained (*E*^*k*^).

To avoid steric clashes, the process checks if *E*^*k*^ has exceeded an admissible threshold. This energy threshold is defined by *ε*_*k*_ and the protein’s potential energy value *E*^0^. In every iteration, *E*^*k*^ value must be below *E*^0^+*E*^0^·*ε*_*k*_. To calculate *ε*_*k*_ the following formula is used:

(6) $$\varepsilon_{k}=\frac{k\cdot \varepsilon }{p}$$

where *ε* determines the maximum change percentage in the protein’s potential energy. As defined before, *p* is the total number of steps of the simulation. If *E*^*k*^ has exceeded the imposed threshold for the current step, the procedure detects the dihedral angle that has generated the higher energy increment. Once detected, the applied rotation is rolled back. The procedure then applies a new rotation with a reduced increment value (*Δψ*_*i*_/2,*Δϕ*_*i*_/2). Again, protein’s potential energy is calculated and checked against the admissible threshold. If the threshold is exceded, the new reduced rotation is undone and the dihedral angle is blocked for the current step. This process is repeated for each dihedral angle of the protein chain.

During the simulation, the number of times each degree of freedom is blocked is saved. When this number reaches an *m* value (user defined), the procedure considers that the dihedral angle cannot rotate in its assigned direction, changing the rotation direction of this dihedral angle (*Δψ*_*i*_=-*Δψ*_*i*_,*Δϕ*_*i*_=-*Δϕ*_*i*_) for the next *n* steps (user defined). Each dihedral angle is independently studied by the procedure, each one with its own record of *m* and *n* parameters.

As the procedure has the capacity to change both dihedral angle increment values and rotation direction, once per step, if a degree of freedom has been blocked, *p* is incremented by 1. This is done with the purpose of better adjusting the energy tolerance of each step which is calculated as a function of *p* (see Eq. (6)).

### Procedure for side chain orientation

**Figure 4:**
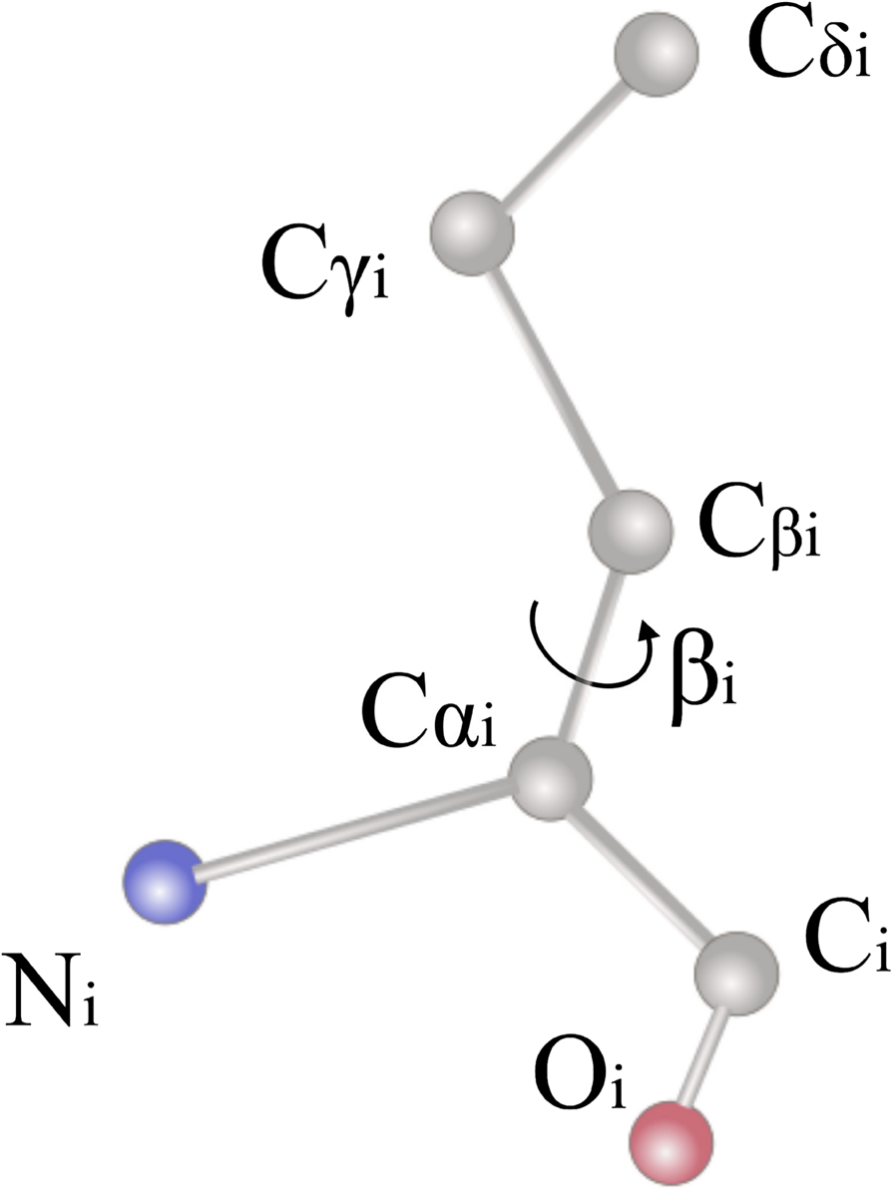
**Side chain *C*** ***α***_***i***_**-*C*** ***β***_***i ***_** bond rotational degree of freedom.**

**Figure 5:**
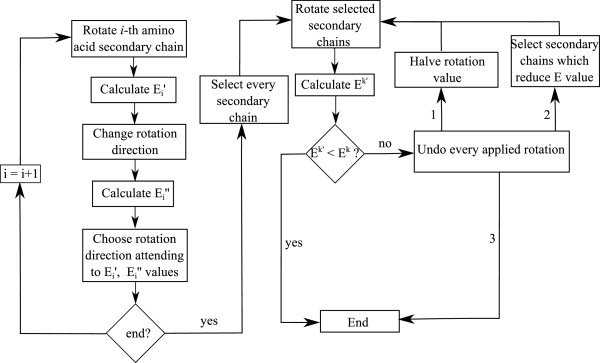
Side chain orientation diagram.

In the strategy on charge of orienting the side chains, the main objective is to achieve a low computational cost. Therefore, in this first stage, only the rotational degree of freedom of *Cα*_*i*_-*Cβ*_*i*_ bonds will be considered (see Figure 4).Another target is to reduce the potential energy value of the protein. This objective may lead to an energy minimization algorithm that could produce an unacceptable increment on the computational cost. To avoid this problem, as it will be explained subsequently the proposed procedure is based on the guidelines shown in Figure 5.

The procedure starts calculating the optimal rotation direction for each side chain. To do so, each side chain is rotated in both possible directions calculating the produced energy increments $E_{i}^{\prime }$ and $E_{i}^{\prime \prime }$ in the process. The selected rotation direction is the one that has produced the higher potential energy decrement, or if both rotation directions have increased the potential energy value, the direction that has produced the lesser energy increment is selected. Accordingly the rotation direction for each side chain is independently defined.

Once the rotation directions are defined, the procedure proceeds to rotate 2° each side chain in its optimal direction. The value has been defined to ensure that no displacement on the edges of long side chains produces steric clashes. After this rotation, it is checked that if the obtained protein’s potential energy $E^{k^{\prime }}$ is lower than the main simulation process produced structure’s one (*E*^*k*^). On the contrary, the process undoes the rotations and new rotation of 1°. Again, obtained protein’s potential energy $E^{k^{\prime }}$ is compared with *E*^*k*^. If again, $E^{k^{\prime }}$ is higher than *E*^*k*^, the process undoes the applied rotations and rotates only those side chains that have produced a decrement on the protein’s potential energy. After the last rotation the values of $E^{k^{\prime }}$ and *E*^*k*^ are checked again, and in the case that $E^{k^{\prime }}$ is higher than *E*^*k*^ last applied rotations are undone, leaving the structure in the initial stage before starting the side chains orientation procedure.

It could be considered unnecessary to validate the last check between $E^{k^{\prime }}$ and *E*^*k*^ since only those side chains that have produced a decrement on protein’s potential energy have been rotated. However, the latter verification must be done because each side chain rotation direction has been independently calculated, without considering the influence of neighboring side chains rotations. A decrement on the potential energy due to the rotation of a single side chain does not ensure that when every other side chain is rotated that decrement is maintained.

### Procedure for secondary structure detection by dihedral angle parameters evaluation

The procedure presented in this paper to detect secondary structures uses the dihedral angle values, which are obtained in any simulation program. A previous step to carry out this strategy is to perform a classification of each amino acid attending to their dihedral angle values as follows: 

• Candidate: an amino acid is considered as a candidate when its dihedral angles are inside a zone of the Ramachandran plot belonging to a secondary structure.

• Stable: an amino acid is considered stable when the procedure has checked that it belongs to a secondary structure.

• Unstable: an amino acid is considered unstable when it cannot be classified as candidate or stable.

**Figure 6:**
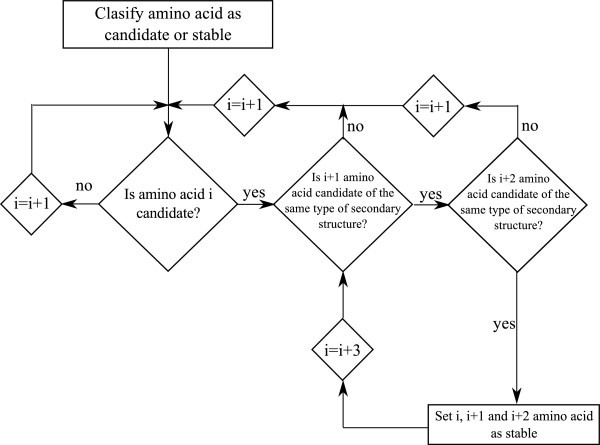
Secondary structure detection process diagram.

To define an amino acid as a candidate, a tolerance has been incorporated with respect to the theoretical values of the dihedral angles. This tolerance has been adjusted to obtain the best possible results, obtaining a final value of 30° for each type of secondary structure.Once the process has classified each amino acid as candidate, stable or unstable, it is possible to start searching for secondary structures. In an initial step, the first candidate amino acid of the protein chain is found and selected. After that, it is checked if the two subsequent amino acids are candidates of the same type of secondary structure. In this case, the three amino acids are classified as stable amino acids. The process continues checking if the next amino acids belongs to the same type of secondary structures, until an unstable amino acid or an amino acid of another type of secondary structure is found. This process is repeated until every amino acid of the protein has been checked. In Figure 6 a diagram of the secondary structure detection process is shown.

The use of a high tolerance resulted in unsatisfactory results, specifically on coil parts of the protein structure. As stated, one of the conditions for an amino acid to be set as a stable amino acid is to be part of a chain of three consecutive amino acids of the same type of secondary structure.This condition reduces the detection of secondary structures on coils of the protein structure, allowing to maintain the tolerance value.

To test the results seven proteins have been analyzed. The proteins under study have allowed us to validate the procedure with different protein sizes and different secondary structure distributions. The obtained results have been compared with each proteins’ structural data, available on the Protein Data Bank (PDB). The results are shown on Table 2.

**Table 2:** Results of the procedure for the detection of secondary structures in the selected proteins

**Protein**	**Molecular mass (Da)**	**% of detected residues in secondary structures**		**% of residues in secondary structures (PDB)**	
		***α*-helix**	***β*-sheet**	***α*-helix**	***β*-sheet**
1zac	9.98	64.4	3.3	70	6
1k9p	10.27	54.4	6.6	60	6
3cln	16.88	54.5	6.2	59	5
1k20	67.75	35.2	24.5	40	20
2peq	30.56	80.5	0	72	0
4fkx	55.13	34.8	19.7	42	16
3sza	104.76	34	22	44	16

**Figure 7:**
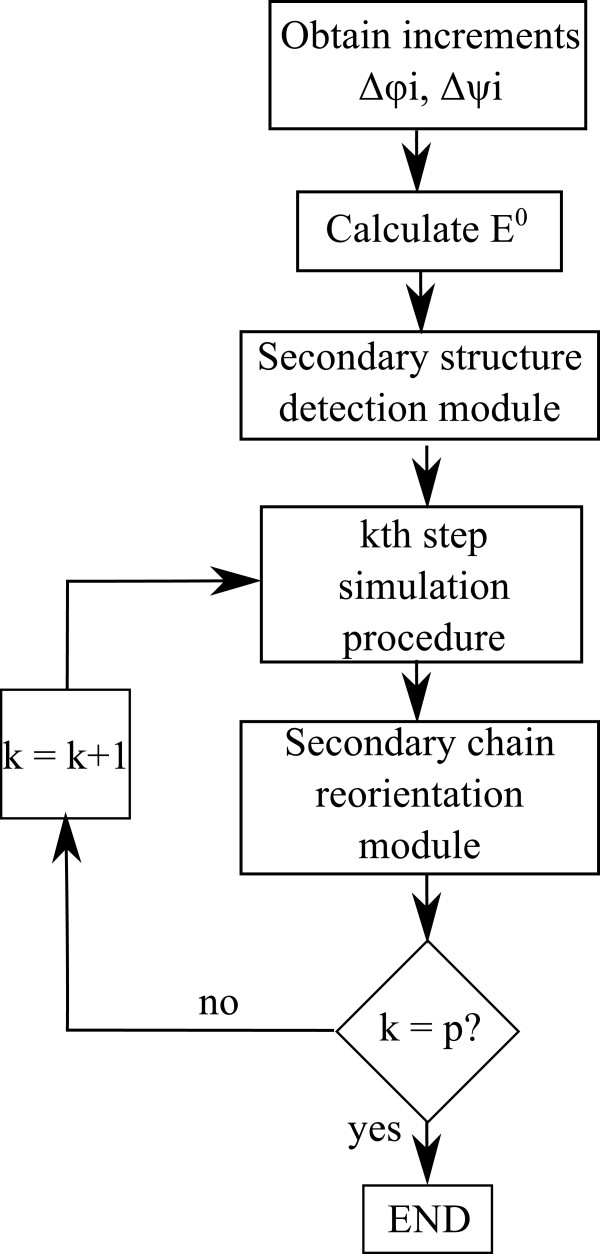
Simulation process diagram including side chain orientation module and secondary structure detection module.

It can be conclude that the strategy requires a very low computational cost, needing only 8*ms* to complete the secondary structure detection on the biggest protein, 3sza. Thus, its implementation on the simulation procedure does not increase the overall computational cost.Once both procedures have been explained, it is essential to assess how to implement them on the simulation process. The aim of developing both processes, side chain orientation and secondary structure detection procedures, is to keep them independent from the main simulation process. This way, if any of them fails to achieve its objective, i.e., detecting no secondary structure or not reducing the protein potential energy value, the main simulation process can continue. For this reason, the proposed sequence of the strategies is shown in Figure 7.As it can be seen in Figure 7. the secondary structure detection procedure is executed prior to the main simulation process. This allows to extract the degrees of freedom already located on secondary structures, therefore, reducing the number of degrees of freedom from the first step. On the other hand, side chains orientation procedure is carried out after the main simulation process. In the simulation process it is required to obtain a valid structure prior to reducing its energy making use of the side chains orientation procedure. If the latter procedure successes on reducing the protein’s potential energy value, then the main simulation process has a higher amount of available energy for the next step.

## Results

**Figure 8:**
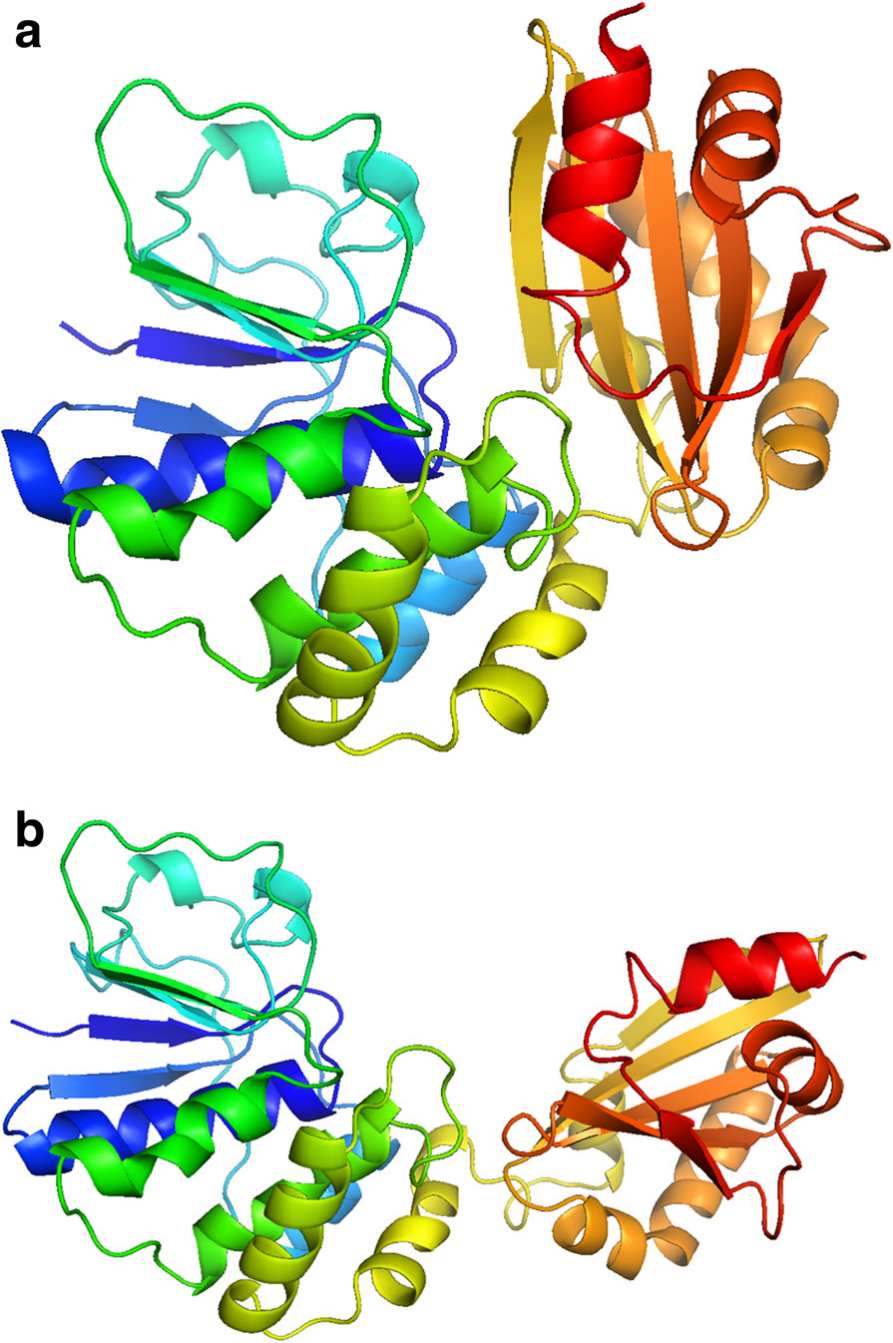
**Initial (a) and final (b) positions of type C inorganic Pyrophosphatase (family II) from Streptococcus gordonii protein.** The movement is given by the aperture of the protein, similar to a crab clamp. Represented with Pymol.

**Figure 9:**
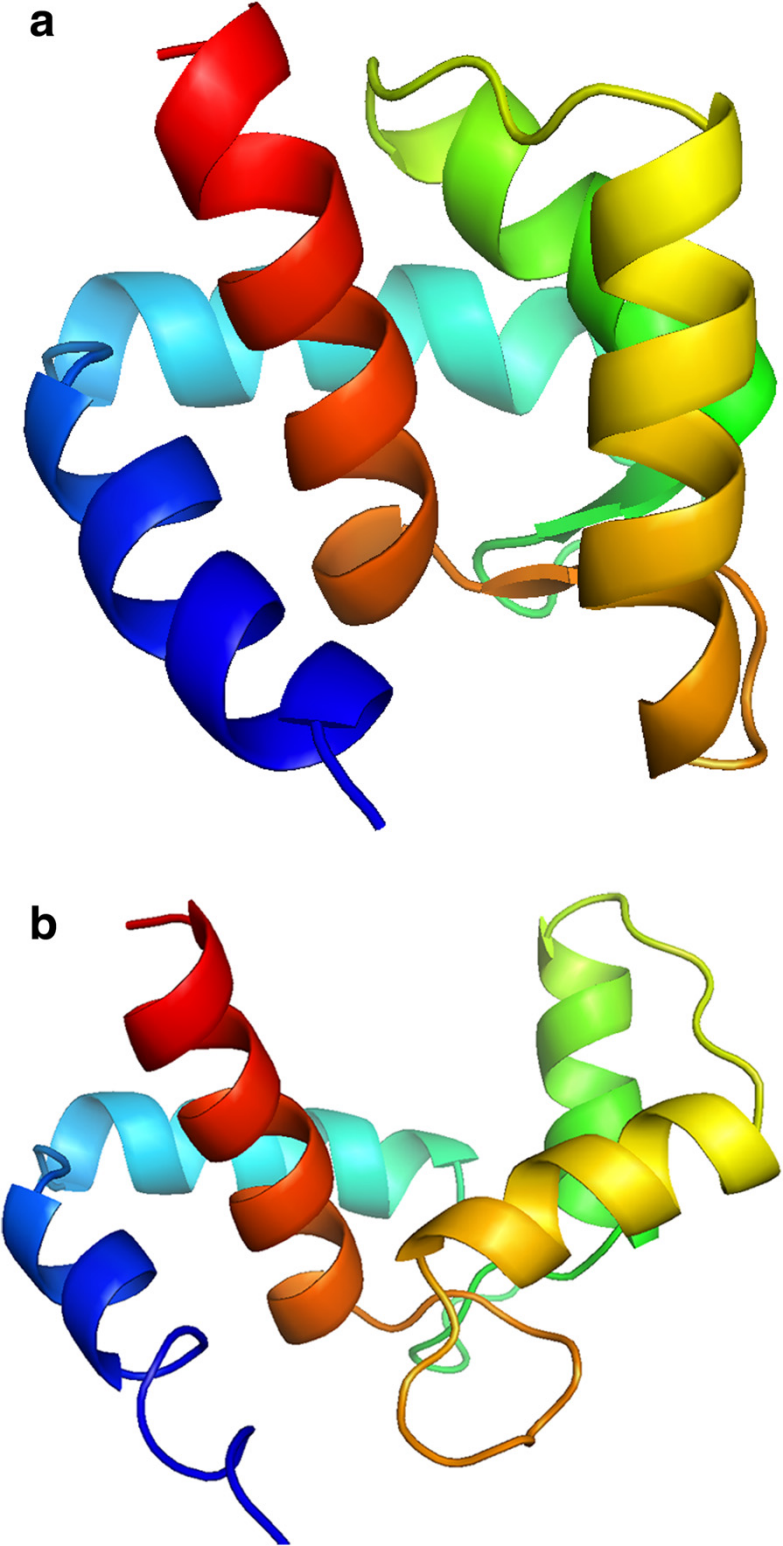
**Initial (a) and final (b) positions of 1zac protein.** The movement is given by displacement of the two helixes of the right part of the protein. Represented with Pymol.

**Figure 10:**
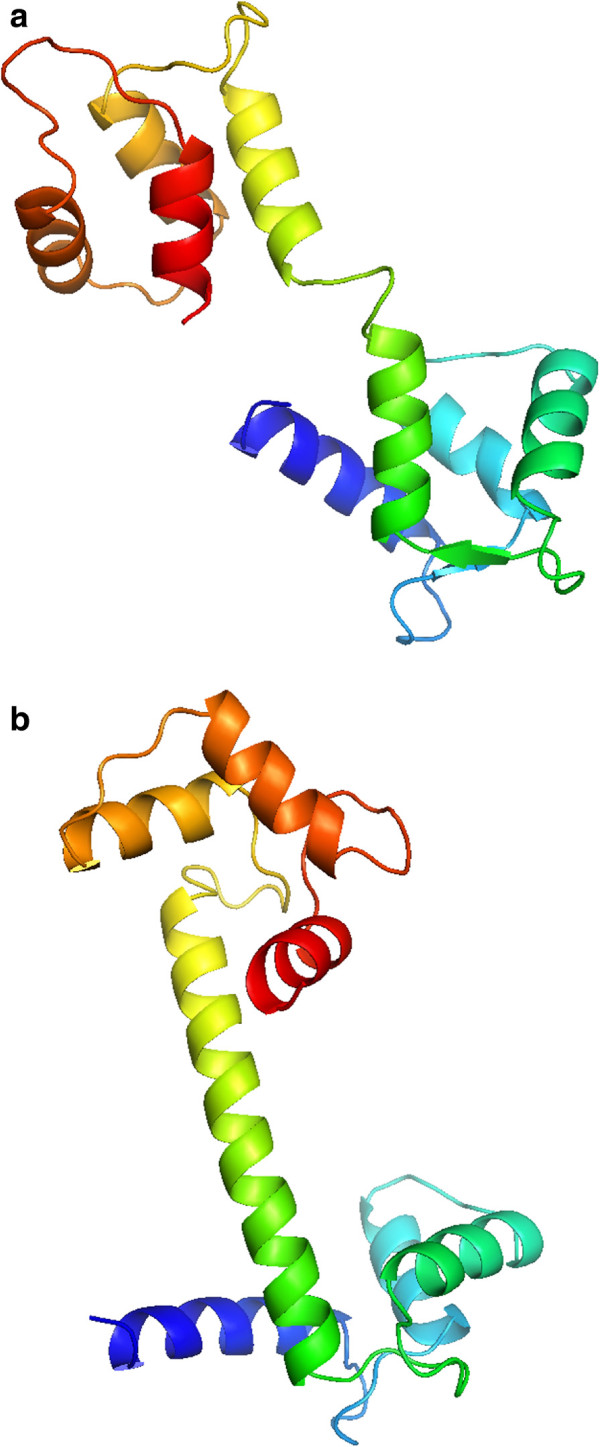
**Initial (a) and final (b) positions of 3cln protein.** The movement is given by the formation of a central *α*-helix. Represented with Pymol.

Each of the simulation strategies will be applied to three different proteins. The first protein, type C inorganic Pyrophosphatase (family II) from Streptococcus gordonii protein, pdb entry 1k20 (see Figure 8). With the proposed modelization, the protein has 4732 atoms with 604 degrees of freedom. The next protein is the Troponin C protein, pdb entry 1zac (see Figure 9). Again, applying the proposed modelization the protein has 1347 atoms and 176 degrees of freedom. Finally, the last protein is the Calmodulin protein, pdb entry 3cln (see Figure 10) with 2201 atoms and 284 degrees of freedom. To test the aforementioned strategies the following type of combinations are proposed: 

• Type 1: General simulation procedure and the side chain orientation strategy will be applied.

• Type 2: The three simulation strategies are applied.

• Type 3: The same as Type 2 but with one exception: the degrees of freedom of side chains located on secondary structures will be blocked.

To select the parameters, the results obtained in previous simulations made with the main simulation process [22,23] have been considered. In particular, the simulation parameters have been set as follows: *p*=100, *ε*=10*%*, *m*=3, *n*=1. Simulations are run in a PC under Windows XP, with a pentium core 2 duo 2.13 GHz making use of a single core.

**Figure 11:**
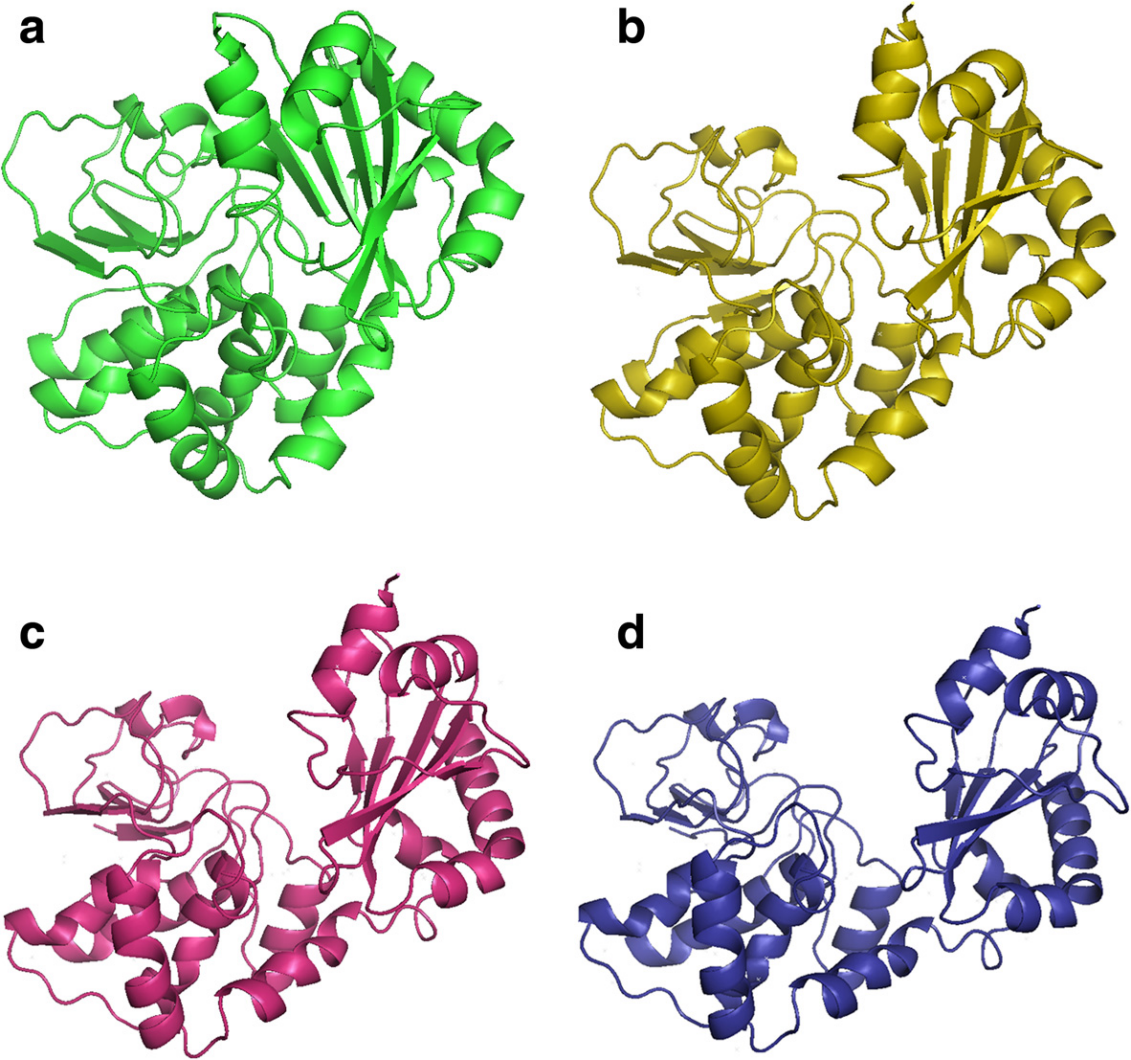
**Snapsots of the simulation of 1k20 protein motion simulation.(a)** Initial position, **(d)** final position and **(b)** and **(c)** intermediate positions.

In Table 3 the results for 1k20 protein are shown. As it can be observed we obtain a minimal rmsd value of 5.97 *Å* with the Type 2 simulation strategy. The Ramachandran plot values indicate that the biological meaning of the protein has been ensured in all simulation strategies. Energy values also indicate the non-existence of steric clashes on the protein structure. Regarding the computational cost of the process, it needs 43 minutes on both Type 1 and Type 2 simulation strategies and 31 minutes on the Type 3 simulation strategy to obtain each intermediate structure of the movement. It can be concluded that the computationally most expensive procedure is the side chain orientation one. This fact is corroborated with the results of Type 3 simulation. In this simulation strategy, the degrees of freedom of side chains located on secondary structures are extracted from the main simulation. In Table 3 it can be seen that the computational time is reduced by 38*%* for each intermediate structure. In Figure 11 several snapshots of the simulation are shown.

**Table 3:** 1k20 Protein results

**Type of simulation**	**rmsd (*Å*)**	**Energy (%)**	**RP (% of atoms inside preferred zones)**	**Step duration**	**Simulation duration**
Type 1	7.4	5.4	93	43 min	44 h
Type 2	5.97	4.4	93	43 min	37 h
Type 3	6.27	5.2	93	31 min	21 h

Results for 1zac protein are shown in Table 4. In this protein minimum rmsd error is obtained on Type 3 simulation strategy with 2.52 *Å*. Again Ramachandran plot values confirm the biological meaning of intermediate structures. Energy values also indicate that steric clashes have been avoided during the simulation procedure. Lastly, regarding the computational cost of the simulation for this protein, the procedure has needed about 2 minutes to obtain each intermediate structure of the motion, completing the simulations in about 80 minutes.

**Table 4:** 1zac Protein results

**Type of simulation**	**rmsd (*Å*)**	**Energy (%)**	**RP (% of atoms inside preferred zones)**	**Step duration**	**Simulation duration**
Type 1	3.15	3.8	98	121 s	64 min
Type 2	3.04	5.9	97	119 s	79 min
Type 3	2.52	4.4	96	97 s	87 min

**Figure 12:**
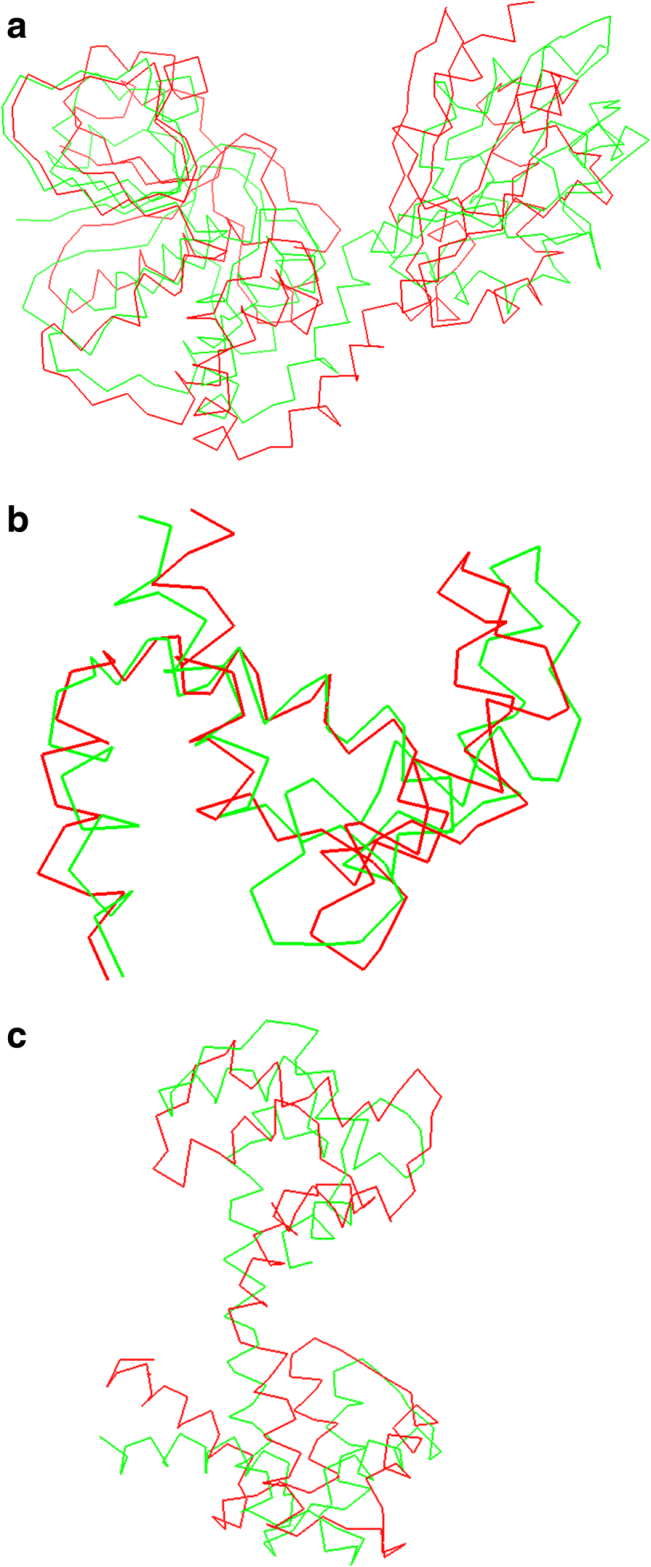
Superpositions of the final structures obtained on the simulation process (red) with the data structures (green) for 1k20 protein (a), 1zac protein (b) and 3cln protein (c).

Finally, the results for 3cln protein are shown in Table 5. The simulation procedure obtains a rmsd error of 6.34 *Å* on Type 1 simulation strategy. Again the Ramachandran plot value ensures the biological meaning of the obtained intermediate structures and energy values indicates that no steric clashes have been produced. As it can be seen, neither Type 2 nor Type 3 simulation strategies are able to achieve a valid simulation. In this particular protein morph, secondary structures degrees of freedom (which have been extracted from the main simulation) are needed to form the central *α*-helix. This fact causes the failure of the aforementioned simulation strategies.The superpositions between obtained final structures (in red) and data structures (in green) are presented in Figure 12.

**Table 5:** 3cln Protein results

**Type of simulation**	**rmsd (*Å*)**	**Energy (%)**	**RP (% of atoms inside preferred zones)**	**Step duration**	**Simulation duration**
Type 1	6.34	2.7	90	301 s	300 min
Type 2	-	-	-	298 s	-
Type 3	-	-	-	225 s	-

## Discussion

The molecular mechanisms underlying the activity of many proteins involve conformational transitions by hinge-bending, which involves the movement of relatively rigid parts of preserved geometry about flexible joints. The detection of both, the rigid domains and the ginger regions, has been largely studied during the last decades [41,42]. The graphical representation of these dynamic events is not only essential to understand how the processes take place, but it helps to unravel mechanistic aspects that are difficult to visualize by less intuitive indirect approaches.

One of the main limitations on the simulation of these conformational transitions is the huge computational cost of actual simulation strategies. Actual approaches struggle to obtain morphs that fulfill both biologic and kinematic requisites. The procedure presented in this paper satisfies both requirements with a good cost-error relation in the studied proteins.

All the procedures presented in the paper make use of the same protein model. In this model bonds are considered as rigid links and the degrees of freedom have been limited to rotations around dihedral angles and each side chain’s first bond rotation. These hypothesis produces a computationally very efficient model of the protein structure with the sufficient mobility to simulate conformational changes.

Current work is focused on the simulation of conformational changes on dimers which posses similar hinge-bending mechanisms. The low computational cost of the presented procedures may be enough to deal with these types of molecules.

## Conclusions

A recurring question in the analysis of molecular mechanisms underlying the regulation of a protein deals with the possible structural routes that let it evolve between two different conformations. The identification of these pathways sets the structural basis for the rational design of molecules that act as modulators (activators or inhibitors) of the proteins activity, becoming an essential tool for the development of new drugs in the pharmaceutical industry.

Few alternatives arise at the time of simulating protein motions. Low computational cost methodologies offer fast results but usually with kinematically senseless trajectories with impossible backbone movements between consecutive positions of the simulation. These methods are based on restrained interpolations of atoms coordinates [40], and rely on intermediate energy minimization processes to solve the steric clashes produced during the simulation. On the other hand, simulation procedures based on molecular dynamics need huge computational resources to complete successful simulations in an acceptable spam of time. Software packages like GROMACS [43,44], NAMD [45], AMBER [46] etc. do not only require shared computing architectures, but due to their complexity its use is limited.

The procedure proposed in this paper offers a fast and reliable method to obtain the motion of the protein. The procedure runs on a single processor and is fond for further improvement by implementing simple distributed computing algorithms. This procedure maintains the kinematic continuity of the movement and ensures the biological sense of the obtained structures.

The presented procedure has been implemented in a new bioinformatic package with the aim of facilitating the comprehension of the processes by which biological machines perform their function. The simulation strategies described herein help the user to understand the behavior of these mechanisms. The described procedures require, at least partially, the availability of the initial and final conformations adopted by the biological machine under analysis. In this regard, the validation indicators implemented in the proposed simulation processes, help to overcome the lack of knowledge in protein structures by providing a modeling tool to reconstruct the fold of a target protein from homologous molecules in other organisms. Moreover, it may also help in deciphering the molecular mechanisms underlying metabolic processes, signaling pathways or transport events, as well as in mapping specific “conformational routes” that characterize the dynamic behavior of a promiscuous protein motif (i.e cystathionine beta synthase (CBS) domains), that undergoes different structural changes upon binding distinct types of ligands (see [47-49]). It should not be neglected the capacity of our software to improve structural search models in molecular replacement methods during the elucidation of novel crystal structures by X-ray diffraction techniques.

## Competing interests

The authors declare that they have no competing interests.

## Authors’ contributions

MD and VP have developed the algorithms; AH has incorporated the kinematic background of serial robots; MD has performed the simulations; MD, VP and LAM-C have analyzed the results; LAM-C has incorporated the biological background for proteins; and MD and LAM-C have written the paper. All authors read and approved the final manuscript.

## References

[B1] Schlick T Molecular Modeling and Simulation An Interdisciplinary Guide, New York: Springer, 1 edition 2006

[B2] Larson SM Snow CD Shirts M Pande VS **Folding@ Home and Genome@ Home: Using distributed computing to tackle previously intractable problems in computational biology** arXiv preprint arXiv:0901.0866 2009

[B3] Voelz VA Bowman GR Beauchamp K Pande VS **Molecular Simulation of ab InitioProtein Folding for a Millisecond Folder NTL9 (1-39)** J Am Chem Soc 2010 132 5 1526 1528 20070076 10.1021/ja9090353 PMC2835335

[B4] Lucas M Encinar JA Arribas EA Oyenarte I García IG Kortazar D Fernández JA Mato JM Martínez-Chantar ML Martínez-Cruz LA **Binding of S-Methyl-52-Thioadenosine and S-Adenosyl-l-Methionine to Protein MJ0100 triggers an open-to-closed conformational change in its CBS motif pair** J Mol Biol 2010 396 3 21 21 10.1016/j.jmb.2009.12.012 20026078

[B5] Kazerounian K **From mechanisms and robotics to protein conformation and drug design** J Mech Des 2004 126 40 45

[B6] Singh AP Latombe J Brutlag DL **A motion planning approach to flexible ligand binding** Proc/ Int Conf Intell Syst Mol Biol; ISMB Int Conf Int Syst Mol Biol 1999 252 261 http://www.ncbi.nlm.nih.gov/pubmed/10786308 10786308

[B7] Hsu D Latombe J Kurniawati H **On the probabilistic foundations of probabilistic roadmap planning** Int J Robot Res 2006 25 7 627 643

[B8] Song G Amato NM **A motion-planning approach to folding: from paper craft to protein folding** Robot Automation, IEEE Trans 2004 20 60 71

[B9] Moll MM Schwarz DD Kavraki LE **Roadmap methods for protein folding** Methods Mol Biol 2007 413 219 239 18075168 10.1007/978-1-59745-574-9_9

[B10] Haspel N Moll M Baker ML Chiu W Kavraki LE **Tracing conformational changes in proteins** BMC Struct Biol 2010 10 Suppl 1 S1 20487508 10.1186/1472-6807-10-S1-S1 PMC2873824

[B11] Enosh A Raveh B Furman-Schueler O Halperin D Ben-Tal N **Generation, comparison, and merging of pathways between protein conformations: gating in K-Channels** Biophys J 2008 95 8 3850 3860 18621834 10.1529/biophysj.108.135285 PMC2553149

[B12] Kavraki LE **Protein-ligand docking, including flexible receptor-flexible ligand docking** 2007 http://cnx.org/content/m11456/latest/

[B13] Cortés J Siméon T de Angulo VR Guieysse D Remaud-Siméon M Tran V **A path planning approach for computing large-amplitude motions of flexible molecules** Bioinformatics 2005 21 1 116 125 http://www.ncbi.nlm.nih.gov/pubmed/15961448 15961448 10.1093/bioinformatics/bti1017

[B14] Raveh B Enosh A Schueler-Furman O Halperin D **Rapid sampling of molecular motions with prior information constraints** PLoS Comput Biol 2009 5 2 e1000295 e1000295 19247429 10.1371/journal.pcbi.1000295 PMC2637990

[B15] Corral J Pinto C Altuzarra O Zubizarreta A **Characterisation of parallel kinematic machines based on structural workspaces** Mech & Ind 2013 14 43 51

[B16] Tirion M **Large amplitude elastic motions in proteins from a single-parameter, atomic analysis** Phys Rev Lett 1996 77 9 1905 1908 10063201 10.1103/PhysRevLett.77.1905

[B17] Atilgan AR Durell SR Jernigan RL Demirel MC Keskin O Bahar I **Anisotropy of fluctuation dynamics of proteins with an elastic network model** Biophys J 2001 80 505 515 11159421 10.1016/S0006-3495(01)76033-X PMC1301252

[B18] Kirillova S Cortés J Stefaniu A **An NMA-guided path planning approach for computing large-amplitude conformational changes in proteins** Proteins 2008 70 1 131 143 http://www.ncbi.nlm.nih.gov/pubmed/17640073 17640073 10.1002/prot.21570

[B19] Kazerounian K Latif K Alvarado C **Protofold: A successive kinetostatic compliance method for protein conformation prediction** J Mech Design (Trans ASME) 2005 127 4 712 717

[B20] Madden C Bohnenkamp P **Residue level three-dimensional workspace maps for conformational trajectory planning of proteins** Int J Robot Res 2009 28 4 450 463

[B21] Kazerounian K Alvarado C Latif K Rodriguez K **Nano-kinematics for analysis of protein molecules** J Mech Des (Trans ASME) 2005 127 4 699 711

[B22] Diez M Petuya V Martínez-Cruz LA Hernandez A **A biokinematic approach for the computational simulation of proteins molecular mechanism** Mech Mach Theory 2011 46 12 1854 1868

[B23] Diez M Petuya V Martínez-Cruz LA Hernández A **Biokinematic Protein simulation by an adaptive dihedral angle approach** MAMT 2013 69 105 114

[B24] Ramachandran GN Ramakrishnan C Sasisekharan V **Stereochemistry of polypeptide chain configurations** J Mol Biol 1963 7 95 99 13990617 10.1016/s0022-2836(63)80023-6

[B25] Jimenez-Roldan JE Freedman RB Römer RA Wells SA **Rapid simulation of protein motion: merging flexibility, rigidity and normal mode analyses** Phys Biol 2012 9 016008 22313618 10.1088/1478-3975/9/1/016008

[B26] Bahar I Atilgan A Demirel M **Vibrational dynamics of folded proteins: significance of slow and fast motions in relation to function and stability** Phys Rev Lett 1998 80 2733 2736

[B27] Schuyler AD Chirikjian GS **Normal mode analysis of proteins: a comparison of rigid cluster modes with Ca coarse graining** J Mol Graph Model 2003 22 3 183 193 14629977 10.1016/S1093-3263(03)00158-X

[B28] Bohnenkamp P Kazerounian K Ilies HT **Structural prediction of peptide based Nano systems via progressive landscape evolution** Proc 12th IFToMM World Congr 2007 1 6 http://www.iftomm.org/iftomm/proceedings/proceedings_WorldCongress/WorldCongress07/articles/sessions/papers/A783.pdf

[B29] Kim MK Jernigan RL Chirikjian GS **Rigid-cluster models of conformational transitions in macromolecular machines and assemblies** Biophys J 2004 89 43 55 15833998 10.1529/biophysj.104.044347 PMC1366543

[B30] Schuyler AD Jernigan RL Qasba PK Ramakrishnan B Chirikjian GS **Iterative cluster-NMA: A tool for generating conformational transitions in proteins** Proteins Struct Funct Genet 2009 74 3 760 776 18712827 10.1002/prot.22200 PMC2930202

[B31] Ansola R Veguería E Canales J Tárrago JA **A simple evolutionary topology optimization procedure for compliant mechanism design** Finite Elem Anal Des 2006 44 53 62

[B32] Lee S Chirikjian GS **Pose analysis of alpha-carbons in proteins** Int J Robot Res 2005 24 2–3 183 210

[B33] Crippen GM Smellie AS Richardson WW **Conformational sampling by a general linearized embedding algorithm** J Comput Chem 1992 13 10 1262 1274

[B34] Cornell WD Cieplak P Bayly CI Gould IR Merz KM Ferguson DM Spellmeyer DC Fox T Caldwell JW Kollman PA **A second generation force field for the simulation of proteins, nucleic acids, and organic molecules** J Am Chem Soc 1995 117 19 5179 5197

[B35] Petuya V Alonso A Pinto C Altuzarra O Hernandez A **A new general-purpose method to solve the forward position problem in parallel manipulators** Adv Robot 2008 22 4 395 409

[B36] Petuya V Gutiérrez JM Alonso A Altuzarra O Hernandez A **A numerical procedure to solve non-linear kinematic problems in spatial mechanisms** Int J Numerical Methods Eng 2008 73 6 825 843

[B37] Ahn S Milner AJ Fütterer K Konopka M Ilias M Young TW White SA **The “open” and “closed” structures of the type-C inorganic pyrophosphatases from Bacillus subtilis and Streptococcus gordonii** J Mol Biol 2001 313 4 797 811 11697905 10.1006/jmbi.2001.5070

[B38] Diez M Petuya V Urizar M Hernandez A **A kinematic approach to calculate protein motion paths** Proceedings of EUCOMES 2009 69 76 http://link.springer.com/chapter/10.1007%2F978-1-4020-8915-2_9

[B39] Ramakrishnan C Ramachandran GN **Stereochemical criteria for polypeptide and protein chain conformations. II. Allowed conformations for a pair of peptide units** Biophys J 1965 5 6 909 933 5884016 10.1016/S0006-3495(65)86759-5 PMC1367910

[B40] Krebs WG Gerstein M **The morph server: a standardized system for analyzing and visualizing macromolecular motions in a database framework** Nucleic Acids Res 2000 28 8 1665 1675 10734184 10.1093/nar/28.8.1665 PMC102811

[B41] Wringgers W Schulten K **Protein domain movements: detection of rigid domains and visualization of hinges in comparisons of atomic coordinates** Proteins 1997 29 1 1 14 9294863

[B42] Kumar S Ma B Tsai CJ Wolfson H Nussinov R **Folding funnels and conformational transitions via hinge-bending motions** Cell Biochem Biophys 1999 31 2 141 64 10593256 10.1007/BF02738169

[B43] Berendsen HJ van der Spoel D van Drunen R **GROMACS: A message-passing parallel molecular dynamics implementation** Comput Phys Commun 1995 91 43 56

[B44] Pronk S Páll S Schulz R Larsson P Bjelkmar P Apostolov R Shirts MR Smith JC Kasson PM van der Spoel D Hess B Lindahl E **GROMACS 4.5: a high-throughput and highly parallel open source molecular simulation toolkit** Bioinformatics 2013 29 7 845 854 23407358 10.1093/bioinformatics/btt055 PMC3605599

[B45] Poghosyan AH Arsenyan LH Astsatryan HV **Comparative NAMD benchmarking on BlueGene/P** MIPRO 2012 319 321 http://ieeexplore.ieee.org/xpls/abs_all.jsp?arnumber=6240663&tag=1

[B46] Pearlman DA Case DA Caldwell JW Ross WS Cheatham TEIII DeBolt S Ferguson D Seibel G Kollman P **AMBER, a package of computer programs for applying molecular mechanics, normal mode analysis, molecular dynamics and free energy calculations to simulate the structural and energetic properties of molecules** Comput Phys Commun 1995 91 1 41

[B47] Ereño-Orbea J Majtan T Oyenarte I Kraus JP Martínez-Cruz LA **Structural basis of regulation and oligomerization of human cystathionine **** *β* ****-synthase, the central enzyme of transsulfuration** Proc Nat Acad Sci USA 2013 110 40 E3790 E3799 24043838 10.1073/pnas.1313683110 PMC3791738

[B48] Baykov AAA Tuominen HKH Lahti RR **The CBS domain: a protein module with an emerging prominent role in regulation** ACS Chem Biol 2011 6 11 1156 1163 21958115 10.1021/cb200231c

[B49] Ereño-Orbea J Oyenarte I Martínez-Cruz LA **CBS domains: Ligand binding sites and conformational variability** Arch Biochem Biophys 2013 540 1–2 70 81 24161944 10.1016/j.abb.2013.10.008

